# Intranasal octenidine and daily octenidine bathing reduces incidence of hospital-onset MRSA bacteremia amongst MRSA carriers

**DOI:** 10.1017/ash.2025.155

**Published:** 2025-09-03

**Authors:** Darius LL Beh, Jia Qi Kum, Glorijoy Tan, Sue Fern Ang, Bee Fong Poh, Brenda Ang

**Affiliations:** 1 Department of Infection Prevention and ControlTan Tock Seng Hospital, Singapore; 2 Department of Infectious DiseasesTan Tock Seng Hospital, Singapore; 3 National Centre for Infectious Diseases, Singapore

**Keywords:** MRSA bacteremia, MRSA carrier, octenidine bathing, intranasal octenidine

## Abstract

**Objectives:** Octenidine dihydrochloride is a topical antiseptic that has been demonstrated to decrease the microbial burden of bacteria colonizing the skin. Various applications of octenidine have been studied with evidence supporting reductions in *Staphylococcus aureus* infections. MRSA carriers are at a higher risk of MRSA bacteremia and can be identified early during admission through screening. This study aimed to evaluate the impact of octenidine on the incidence of hospital-onset (HO) MRSA bacteremia amongst MRSA carriers. **Methods:** A quasi-experimental before-and-after interventional study was conducted in a single 1700 bed academic teaching hospital. From December 2021 onwards, five days of intranasal octenidine and daily octenidine bathing until discharge was introduced only for MRSA carriers. Screening for MRSA colonization status occurs on admission (either by nasal PCR or nasal, axilla and groin culture, and upon inpatient transfer or through clinical cultures. An HO-MRSA bacteremia event is defined as occurring more than 3 days from admission or more than 14 days from the last positive date. Baseline yearly incidence of HO-MRSA bacteremia for 2021, and the proportion occurring in MRSA and non-MRSA carriers was determined. This was then compared to yearly incidence observed post-intervention in 2022 and 2023. **Results:** Between 2021-2023, the yearly incidence of HO-MRSA bacteremia in carriers decreased steadily from a baseline of 11 to 10 and then to 6 per 1000 patients. In contrast, over the same period the yearly incidence in non-MRSA carriers initially increased from a baseline of 0.26 to to 0.33, before decreasing to 0.22 per 1000 patients. Overall yearly incidence decreased by a greater proportion in those receiving octenidine compared to those who did not – 45.5% vs 15.4%. **Conclusion:** Intranasal octenidine and daily octenidine bathing when performed on a high risk group such as MRSA carriers, reduces the incidence of HO-MRSA bacteremia.

